# Employers should promote smoking cessation in the workplace

**DOI:** 10.1177/17579139241264177

**Published:** 2024-08-30

**Authors:** H Blake

**Affiliations:** Professor of Behavioural Medicine, School of Health Sciences, University of Nottingham, Nottingham, UK; NIHR Nottingham Biomedical Research Centre, Nottingham, UK

## Abstract

This feature article explores the role of the workplace in smoking cessation. Blake looks into the current measures in place, and explores how employers can benefit from and support employees by implementing smoke-free policy and regulation in the workplace.


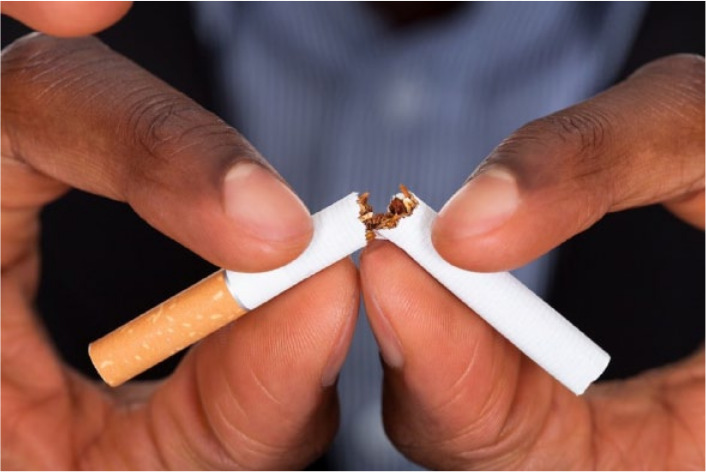


Smoking is a leading cause of preventable death and chronic disease, associated with high socioeconomic burden.^1^ Tobacco control is therefore a key part of national and international public health policy.^2,3^ The World Health Organization (WHO)^3^ Global Action Plan for the Prevention and Control of Noncommunicable Diseases 2013–2020 includes a target for reducing the global prevalence of tobacco use by 30% by the year 2025, relative to 2010. In 2019, in the green paper ‘Advancing our Health: prevention in the 2020s’,^4^ the government announced an ambition for England to become ‘smokefree’ (defined as adult smoking prevalence of 5% or less) by 2030. Implementing smoke-free policies and improving access to smoking cessation advice and support is a vital part of achieving this.

In England, legislation was introduced in 2007, which made it illegal to smoke in all public enclosed or substantially enclosed areas, including workplaces.^5^ Legislative smoking bans have benefits for population health. A Cochrane review (71 studies, 21 counties) found moderate-quality evidence that countries which enact national legislative smoking bans have improved health outcomes, specifically cardiovascular disease, through a reduction in secondhand smoke. There was low-quality evidence of reduced mortality for smoking-related illnesses.^6^

The workplace is an important setting for health promotion. Health and wellbeing at work is specified as an organisational priority in the National Institute for Health and Care Excellence (NICE)^7^ Quality Standard on Healthy workplaces (Quality Statement 1). Around three-quarters of the UK population participate in the labour force,^8^ which makes workplaces an ideal venue for reaching large numbers of people for health promotion intervention. This may include smoke-free workplace policies, and smoking cessation advice and support. Smoke-free environments benefit the whole workforce; those who smoke, and others around them, through reduced or removed exposure to secondhand smoke. There may be particular health benefits of smoke-free workplaces for groups most at risk, such as those who are pregnant, and those with chronic conditions like asthma, respiratory diseases, diabetes, chronic kidney disease, or heart disease. Including tobacco control in workplace health promotion has the potential to make a modest contribution to reducing health inequalities by reaching demographics who may have a higher prevalence of smoking and/or exposure to secondhand smoke (e.g. blue-collar workers), and those who are under-served by health promotion or are from marginalised groups (e.g., men, blue-collar workers, people with disabilities, minority ethnic groups, economic migrants).

Regarding the workforce, studies have found that workers view smoke-free policies positively, albeit employees believe that actions are needed by employers to enforce them.^9^ Workplace smoke-free policy may lower exposure to secondhand smoke, reduce smoking behaviours, and raise awareness about smoking harms.^10^ The type of interventions found to increase the likelihood of abstinence in workplace settings are individual and group counselling, pharmacological treatment for nicotine addiction, and interventions that target smoking cessation as the primary or only outcome.^11^ However, smoking cessation programmes at work have been found to be most effective for those for whom stopping smoking is already a personal priority (i.e. those who have moved past the contemplation stage, into the action stage).^12^ Furthermore, there may be challenges in reaching some types of workers with workplace health intervention, who may be at increased risk of tobacco exposure, such as those in precarious work (defined as work instability), those with a, lack of labour protection, job insecurity, and social and/or economic vulnerability.^13^

Critically, there is a need to convince employers, with limited resources, of the value of embedding smoke-free policies, tobacco campaigns, and smoking cessation interventions within organisations. Employer-facing professional bodies and networks should therefore highlight the business case for investment of time and resources in this aspect of workforce health and provide case examples of best practice from different types of organisation.

In terms of the business case, there is a strong narrative around workplace health promotion as a corporate social responsibility. It is argued that employers have societal responsibilities that go beyond the economic arguments about cost reduction and maximising profit.^14^ Nonetheless, there are strong economic arguments for health promotion endeavours by employers. Organisations incur indirect costs (impacts on workplace absenteeism and productivity) for smoking employees.^15^ Smoking contributes significantly to ill-health, and employee ill-health is extremely costly. In 2023, 36% of working age people were living with more than one long-term condition, with over 2.5 million people economically inactive because of long-term sickness.^16^ Overall, an industry poll of employees, commissioned by Zurich UK for a joint report for the Centre for Economics and Business Research (CEBR),^17^ found that work absences due to long-term sickness cost the economy around £32.7bn in lost productivity in 2023, estimated to rise to £66.3bn per year by 2030. Smoking makes a significant contribution to these costs. There is robust evidence from a systematic review with meta-analysis, to show that smoking increases both the risk and number of sickness absence days in working populations. Specifically, people who smoke have a 31% increased risk of sickness absence, and 2.89 more sickness absence days per year compared to people who do not smoke, regardless of geographical location, gender, age, or occupational class.^18^ Although there is heterogeneity in the economic measurement of smoking cessation interventions, workplace smoking cessation is broadly thought to be cost-effective. As one example, an economic analysis of a workplace smoking cessation programme calculated a return-on-investment (ROI) of 15.39.^19^ This meant that the economic effect (reduction in productivity costs and medical expenses) was 15.39 times the cost of implementing the programme. Promoting smoking cessation advice and support therefore makes economic sense to employers.

Equipped with the business case, employers need guidance on the steps to take to promote smoking cessation in the workplace. Guidance on tobacco control is available from the National Institute for Health and Care Excellence (NICE),^2^ which include specific recommendations for employers. This advice includes publicising local and/or onsite treatment for tobacco dependence, allowing workers to attend services offering treatment for tobacco dependence during their working hours without loss of pay, and negotiating a smoke-free workplace policy together with workers or their representatives. Policy might include signposting to support and treatment for tobacco dependence, establishing smoke-free policy and regulations around smoking breaks at work. However, research is needed to explore employers’ willingness to implement these guidelines within organisations of different sizes, types, and sectors. Research could explore the barriers and enablers of implementation across organisations with different employee profiles, and across different employment settings and contexts, such as small-to-medium enterprises which may experience unique challenges in the provision of workplace health promotion due to resource limitations.

In summary, all efforts to reduce smoking will contribute to national and international public health priorities. Forward-thinking employers have workforce health and wellbeing embedded firmly within organisational policy and practice. As a social responsibility to contribute to improving population health, and as a business priority, to reduce the economic impact of workforce ill-health.
